# Assembling networks of microbial genomes using linear programming

**DOI:** 10.1186/1471-2148-10-360

**Published:** 2010-11-20

**Authors:** Catherine Holloway, Robert G Beiko

**Affiliations:** 1 Faculty of Computer Science, Dalhousie University, 6050 University Avenue, Halifax, Nova Scotia, B3 H 1W5, Canada; 2 Department of Physics and Atmospheric Science, Dalhousie University, Halifax, Nova Scotia, B3 H 3J5, Canada; 3 Institute for Quantum Computing, University of Waterloo 200 University Ave. West, Waterloo, Ontario, N2L 3G1, Canada

## Abstract

**Background:**

Microbial genomes exhibit complex sets of genetic affinities due to lateral genetic transfer. Assessing the relative contributions of parent-to-offspring inheritance and gene sharing is a vital step in understanding the evolutionary origins and modern-day function of an organism, but recovering and showing these relationships is a challenging problem.

**Results:**

We have developed a new approach that uses linear programming to find between-genome relationships, by treating tables of genetic affinities (here, represented by transformed BLAST e-values) as an optimization problem. Validation trials on simulated data demonstrate the effectiveness of the approach in recovering and representing vertical and lateral relationships among genomes. Application of the technique to a set comprising *Aquifex aeolicus *and 75 other thermophiles showed an important role for large genomes as 'hubs' in the gene sharing network, and suggested that genes are preferentially shared between organisms with similar optimal growth temperatures. We were also able to discover distinct and common genetic contributors to each sequenced representative of genus *Pseudomonas*.

**Conclusions:**

The linear programming approach we have developed can serve as an effective inference tool in its own right, and can be an efficient first step in a more-intensive phylogenomic analysis.

## Background

Although lateral genetic transfer (LGT) has been recognized for many decades as a potentially important force driving the evolution of prokaryotes [1-3], only in the genome sequencing era has its frequency and importance been fully appreciated [4-6]. LGT mediated by processes of DNA transfer and recombination occurs within populations of closely related bacterial strains [7,8], can operate at great phylogenetic distances [9-11], and can affect bacteriophage, viruses, protists and multicellular eukaryotes [12]. Some observed LGT events are obviously transient and likely part of a cycle of saltation and purging [13], while others confer clear adaptive advantages to their host, and have become fixed in a set of descendent lineages [14,15]. The principal evolutionary consequence of LGT is that traits do not need to be invented *ab initio *within a genome through (for example) neofunctionalization of a paralogous sequence, but can instead be rapidly acquired from another organism and integrated into the host regulatory and metabolic apparatuses [16]. Grasping the nature and extent of LGT is fundamental to our understanding of evolution, but at the same time is essential if we are to understand how specific microorganisms and communities of microorganisms can change in response to new environmental challenges and opportunities.

The inference of historical events from modern genome sequences is a challenging task that has spurred the development of many phylogenetic and non-phylogenetic methods. Technical problems include defining models of sequence similarity and evolution, choosing a sequence data set of interest (which is often much less than the entire suite of genes from a set of genomes), and implementing thresholds that demarcate vertical, lateral, and 'uncertain' relationships: each of these will influence the recovered frequency of LGT, the types of genes implicated, and the genomes that preferentially share genes. The very definition of 'vertical' vs. 'lateral' requires that some component of the evolutionary signal recovered from a set of genes be accorded privileged status, tracking a Tree of Life or, more weakly, a tree of cellular divisions. Decisions on which signals are to be treated as vertical are often based on a plurality consensus or a subset of core genes that are thought to be recalcitrant to transfer [17-20].

The problems with using an aggregated or consensus signal to define vertical inheritance are twofold. First, while overwhelming support for a particular relationship might be taken to indicate vertical inheritance, the phylogenetic signal becomes less clear as deeper and deeper relationships are examined, to the point where little can be confidently said about the relationships among bacterial phyla, either in phylogenetic or phylogenomic terms [21]. Second, in any case where conflicting phylogenetic signals are combined, the obtained consensus may reflect phylogenetic averaging artifacts that capture none of the input evolutionary signals [22,23]. In such cases, relationships that appear to be lateral in nature may in fact be vertical or vice versa, or the entire set of implied evolutionary connections may be invalid. A frequently cited example of this is the thermophilic bacterium *Aquifex aeolicus*, which has been described as an early-branching bacterium with similarities to *Thermotoga *and other thermophiles including many Archaea [24,25], or as an unusual Proteobacterium with strong LGT connections to other thermophiles [26,27]. Detailed analysis of many phylogenetic trees can reveal the complex relationships of such lineages, whereas aggregation of these signals conflates the set of affinities they have to other lineages. However, alignments of single genes or proteins may not harbor enough information to recover evolutionary relationships with high confidence.

In spite of these limitations, building a comprehensive profile or map of genetic affinities is a worthwhile goal. Recent efforts have characterized phylogenetic discordance without reference to a 'central tendency' tree. Puigbò et al. [11] searched for trends of phylogenetic consistency, first among 102 large, 'nearly universal' trees and then using several thousand trees with no taxonomic restriction, and found rampant discordance but also a statistically supported central trend of concordance. Consistent with previous work [5,21], concordance was highest among closely related lineages, whereas little or no consistency was found in the deeper relationships within Bacteria and Archaea. Susko et al. [28] used a heatmap approach to characterize the consistency of relationships within the Gamma-proteobacteria, and found weak phylogenetic agreement within the data set. However, their results also highlighted a pitfall of many analyses: the majority of genes failed to strongly support any relationship, and treating these genes as if they agreed with the plurality signal would overestimate the support for a single evolutionary path. Lima-Mendez et al. [29] developed a novel approach for bacteriophage genomes based on Markov clustering and graph reconstruction, with nodes representing individual phage genomes and edges representing genes common to both linked genomes. Phage relationships are not generally thought of in terms of a phylogenetic tree, and indeed the authors were aiming to define coherent 'modi' [30] for multidimensional classification. It is perhaps natural that such a scheme, which presumes nothing about the laterality or verticality of relationships, was first developed for phage genomes. More recently, a similar network approach was applied to gene sets from > 100 prokaryotic chromosomes and > 100,000 vector (phage and plasmid) sequences, with analysis of graph connectivity suggesting that plasmids and not phage are key elements linking genomes [31].

While networks that capture all affinities or the best affinities of all genes in a genome can usefully display lateral connections, it is worth considering the entire spectrum of relationships for each gene under consideration. This can be important if, for instance, the best few BLAST matches to a given query protein are statistically indistinguishable. We have developed an approach to analyze and visualize the affinities among genomes which is based on linear programming (LP). This algorithm considers the relative strength of different matches and aims to recover distinct affinities of different members of a set of genomes. For each genome in turn, the method uses a series of similarity scores (here, tables of BLASTP scores between the candidate genome and the full set of microbial genomes) to construct a weighted, directed graph which we term an *intergenomic affinity graph *with large connection weights reflecting strong affinities between genomes. The use of a directed graph allows us to observe asymmetric relationships in which a given genome A has contributed genetic material to another genome B, but not vice versa. We first demonstrate the utility of our method by benchmarking on genome data simulated under various regimes of LGT, then apply it to two distinct sets of microorganisms: a set of thermophiles with affinities to *A. aeolicus*, and the Pseudomonads, a group with highly plastic genomes that can thrive in many habitats.

## Results

We start this section by defining the intergenomic affinity graph, and introducing the idea of 'comparison genomes' that are used as the building blocks for this graph. Following this, we formulate several candidate techniques for computing comparison genomes, several variants of which are based on LP. Details of the parameter settings used for BLAST and EvolSimulator can be found in the Methods section.

### Intergenomic affinity graphs and optimal comparison genomes

An intergenomic affinity graph G for a set of genomes C = {C_1_, C_2_, ..., C_m_} is defined as a set of vertices V = {V_1_, V_2_, ..., V_m_}, each corresponding to a genome in set C, and a set of edges E. An edge from vertex V_1 _to vertex V_2 _implies that a subset of C_1_'s genome has an affinity to a subset of C_2_'s genome that is relatively strong in comparison to other members of C. A vertex may have multiple outgoing edges if it has strong affinities to several other genomes, or it may have a single outgoing edge if a single other genome in C is its strongest genetic partner. The latter case might arise, for example, if genome C_i _shares a recent common ancestry with genome C_j_, and has not been impacted by LGT since that divergence. Edges in G are directed because relative affinities are not guaranteed to be symmetric; C_i _may be a significant contributor to C_j_, but not vice versa. Edges carry weights that reflect the relative contribution of different genomes to the target genome; outgoing edges from a genome are normalized to sum to 1.0.

The intergenomic affinity graph shows a complete set of genetic relationships among a set of genomes, but the graph formulation is based on a genome-by-genome approach to identifying genetic similarities. The gene content of a genome C_i _is evaluated against all other genomes to identify elements (genes or sets of genes) of these genomes that are highly similar to elements of C_i_, and therefore represent likely contributors to the gene content of C_i_. The union of these contributing elements defines the *optimal comparison genome *(OCG) for C_i_; the OCG may contain components of one, several, or all members of the set C - C_i_. In the intergenomic affinity graph, the set of edges leading to vertex V_i _and their relative weights reflect the relative contribution of other genomes to the OCG. The composition of the OCG and the evolutionary interpretation of the affinity graph depend on the choice of function used to build the OCG; ideally the graph should capture all significant vertical and lateral evolutionary relationships that exist in a set of genomes. We introduce several alternative functions in the next section.

### Techniques for constructing optimal comparison genomes

LP is a method of maximizing or minimizing a linear equation (called the objective function) subject to a set of constraining linear equations and inequalities. Previous applications of LP in bioinformatics include gene and species tree reconstruction [32-34] and protein structural inference via threading [35]. Our LP formulation is intended to capture distinct affinities between a reference genome X consisting of *n_0 _*genes X_1_, X_2_, ..., X_n_, and a set of *m *comparison genomes Y = {Y_1_, Y_2_, ..., Y_m_}, each consisting of *n_j _*genes e.g. Y_1,1_,Y_1,2_, ... Y_1,n1_. The basis for identifying relationships is an *n *× *m *similarity matrix A, with each row A*_i._*corresponding to protein-coding gene *i *from X, and each entry A*_ij _*representing the similarity between gene *i *and the best-matching gene *g_j _*from Y_j_, 1 ≤ *j *≤ *m*. Each row A*_i. _*therefore constitutes a weighted phylogenetic profile [36] that captures the relative genetic affinity of X*_i _*for each member of Y. The aim of our approach is to construct the OCG for X from members of set Y. Our initial analyses use a transformed matrix of BLASTP results, with

(1) $$A_{i,j}=-\log(\alpha_{ij})$$

where α_i,j _is the expectation value of the BLASTP score with *x_i _*as query and *g_j _*as subject.

A simple approach to inferring genomic affinities from these BLASTP scores would be to directly use matrix A to identify incoming edges to X in the intergenomic affinity graph, with weights *w_j _*computed as follows:

(2) $$w_{j}=\frac{\sum_{i=0}^{n_{0}}A_{ij}}{\sum_{i=0}^{x_{0}}\sum_{k=1}^{m}A_{ik}}$$

Repeating this procedure using each genome in (Y ∪ X) as the reference genome in turn would produce an intergenomic affinity graph showing the complete set of genetic affinities between all pairs of genomes. We term this approach to generating *w_j _*the *weighted maximum (wmax) *technique.

However, such an approach ignores the potential for repeated structure in different rows of the BLAST table: for example, if two genomes Y_1 _and Y_2 _are both closely related to genome X, with Y_1 _and X as sisters and Y_2 _a close outgroup to the other two, then we would expect to see many rows in the transformed BLAST table where *A_X1 _*is only slightly greater than *A_X2_*. Based on these rows, *wmax *would assign weights such that *w*_Y1 _>*w*_Y2_, but with *w*_Y1 _only slightly greater than *w*_Y2_.The averaging effect of *wmax *does not distinguish between the case above, where *A_X1 _*is consistently if only slightly greater than *A_X2 _*in many rows, versus a situation where *A_X2 _*is sometimes greater and sometimes less than *A_X1_*, but the mean affinity for Y_1 _is greater than that for Y_2_. To distinguish these cases, we need an approach that emphasizes the consistently greater similarity of partner genome Y_1 _in a weighted network of genome affinities. If we consider construction of the comparison genome as an optimization problem on the set of genetic affinities in A, we can formulate an LP approach to find the optimal weighting of genomes in Y with respect to X that achieves this aim. The goal of the strategy is to maximize ε and choose *w_j _*such that:

(3) $$\sum_{j\in Y}w_{j}=1$$

(4) $$w_{j}\geq 0\;\forall \;j\in Y$$

(5) $$A_{1j}w_{1}+...+A_{ij}w_{i}+...+A_{mj}w_{m}\geq \varepsilon \;i\leq j\leq m.$$

A given *w_j _*value will be applied uniformly to the corresponding column A_.j_, and therefore allow A_ij _terms to contribute to the sum (which must be ≥ ε for each row, due to the constraint that (5) imposes) even when they are not the maximum term from that row.

One potentially misleading implication of the above approach is that rare genetic affinities, i.e. X_i _genes with best matches to unusual target Y_j _genomes, can lead to a very high *w_j_*, since the sum of scores for X_i_*'*s row is still constrained to be ≥ ε. A large *w_j _*in this situation is indicative of very distinctive matching patterns such as an LGT connection between distantly related genomes. To better quantify the relative abundance of different relationships to the various *X_i_*, we multiply ε for each row *i *by the sum of *A_i,j _*for all reference genomes *j*. As a consequence, the limiting constraints will be those with the greatest total distance scores. This reweighting will increase *w_j _*values for Y_j _genomes with many strong affinities to X, and will diminish but not eliminate rare affinities. We refer to this reweighting approach as the *rLP *strategy.

As an alternative to reweighting, we also considered the application of thresholds to matrix A prior to computing the LP solution. In this scenario, any *A_i,j _*< threshold *T *was set to zero, with the effect that any BLAST matches with e-value > 10*^T ^*are ignored. We refer to this as the *tLP-T *strategy, where *T *= the chosen threshold.

Finally, we considered a strategy in which proteins we defined as 'dominated' were removed. Given two proteins from the query genome, *p_1 _*and *p_2_*, we say that *p_1 _dominates p_2 _*if *A_1,m _*≥ *A_2,m _*for all target genomes *m*. The removal of dominated proteins yields a reduced set that includes (i) proteins from X that constitute the best overall matches to each of the genomes in Y; and (ii) proteins that are not in set (i), but nonetheless have sufficiently distinct match profiles that they are dominated by no single protein in set (i). This strategy emphasizes slowly evolving proteins (since their *A_i,j _*entries will tend to be larger) and is expected to highlight particularly strong (vertical and frequent lateral) connections between X and members of Y. The strategy is analogous to that employed by others [18,37] to isolate genes that are particularly informative, although our criteria are different, particularly in that we do not require all retained genes to exhibit the same distribution pattern or phylogenetic history. This final strategy is referred to as *ndLP*.

All LP approaches outlined above have a total of *m *+ 1 variables to optimize, with one variable per genome in Y in addition to ε. The maximum number of constraints for the problem is equal to *m *(a nonnegativity constraint for each genome weight) + *n_0 _*(the set of constraints imposed by each gene's phylogenetic profile) + 1 (since all genome weights must sum to 1.0). Fewer constraints will exist if the number of genes under consideration is less than *n_0 _*due to some genes having no BLAST matches, and/or genes being removed by the *ndLP *strategy. The procedures described above will produce a set of weighted edges originating from genome X and terminating in a subset of genomes in Y. To build the intergenomic affinity graph, we repeat the analysis for each genome in (Y ∪ X).

### Validation on simulated genomes

We first evaluated the efficiency of the LP approach on data with a similar structure and magnitude to BLAST comparisons of genomes. The simplex method we use for optimization is exponential with respect to the number of inputs, but runs in polynomial time in the average case [38]. For genomic data sets of size *k = *2 to 40 in increments of 2, we generated affinity tables with 3000 rows and *k *- 1 columns. Each table entry was filled with a random number. We then applied the *ndLP *and *rLP *techniques using the glpk solver (see Methods) to these tables and evaluated the total running time. The scaling of runtime (Supporting Figure S1 in Additional File 1) with input number of genomes was slightly greater than linear in the number of input genomes, with times < 0.01 seconds for *k *≤ 10, up to 0.0835 seconds for *k *= 40. This is a tiny fraction of the time needed to generate BLAST results for a comparable number of microbial genomes, and suggests that the time taken by the LP solver will not be limiting in the analysis of datasets with *k *much greater than size 40.

EvolSimulator [39] is a program that simulates the evolution of genomic lineages via processes of speciation and extinction. Each EvolSimulator run starts with a single genome containing an ancestral set of genes; as lineages diverge, gene content can change via duplication, loss, and LGT, with different user-specified models of LGT available. We used EvolSimulator to generate populations of up to 50 genomes (see Methods for details) with different regimes of LGT influencing the success of attempted transfer events for a given donor-recipient pair. The simplest scenario allowed no gene content change whatsoever (*noLGT-noLoss*), so all genomes at the end of this simulation contained a single copy of each ancestral gene that was modified through mutation and substitution events. All other scenarios allowed genome sizes to change via duplication or loss at each step of the simulation. A single simulation was run without LGT, but with the possibility of gene duplication and loss (*noLGT*). Three separate regimes of LGT were then imposed in separate sets of runs: (i) unconstrained or 'random' LGT (*randLGT*), where any donor-recipient genome pair is equally likely to participate in LGT; (ii) divergence-restricted LGT (*divLGT*), in which the probability of successful transfer decreases with increasing divergence (i.e., number of iterations since the most common recent ancestor); and (iii) habitat-restricted LGT (*habLGT*), in which genomes occupy specific habitats and niches with rare switching events, and transfers can occur only between genome pairs that occupy the same habitat at a given iteration. For each regime, we varied the intrinsic rate at which LGT events were proposed by two orders of magnitude in three separate runs. All combinations of regime and rate were repeated 50 times, except for the habitat simulations which were replicated 250 times each.

Since EvolSimulator records the complete history of all genomes in a given simulation, we were able to evaluate our network inference strategies in the 'vertical' context of known speciation histories, in light of known LGT events, and with knowledge of currently assigned genomic habitats and complete habitat histories. We devised statistics that characterize the structure of graph G in light of the known connections between genomes.

The **treeness **score T expresses the match between the reconstructed evolutionary network and the simulated genome tree:

(6) $$T=\sum_{i}\sum_{j}w_{i\to j}\times \left(1.0-dist_{i\to j}/dist_{\max}\right)$$

where *i *and *j *are two genomes for which a connection *i *→ *j *is defined, *w*_*i→j *_is the edge weight from *i *to *j*, *dist_i→j _*is the number of internal nodes in the reference tree that are traversed by the shortest path from leaf *i*'s closest parent (internal node) to leaf *j*'s closest parent in the reference tree, and *dist_max _*is the largest such distance in the entire tree. The assignment of relatively large weights to siblings in the tree (*dist_i→j _*= 0) will minimize the ratio of distances in (6), leading to large overall values for *T*. Since the treeness score scales with the number of genomes, we divided *T *by the total number of genomes considered to yield a normalized treeness score, *T_norm_*, with range 0.0 <*T_norm _*≤ 1.0.

The **habitat **score H is defined as:

(7) $$H=\sum_{i}\sum_{j}w_{i\to j}\times \left(dist_{i\to j}/dist_{\max}\right)\times Z_{i,j}$$

with *Z_i,j _*an indicator function which is equal to 1.0 if *i *and *j *are in the same habitat and 0.0 otherwise. Since we set habitat changes to occur relatively infrequently in our simulations, habitat similarity and evolutionary history were conflated to a large degree. To minimize the impact of this conflation and to emphasize the detection of habitat-directed LGT connections between distant relatives, we use the raw distance ratio (i.e., not subtracted from 1.0) to maximize the contribution of distantly related genomes from the same habitat. As with the treeness scores, we divided *H *by the number of genomes to yield the normalized habitat score *H_norm_*, with range 0.0 ≤ *H_norm _*≤ 1.0.

**Figure 1:**
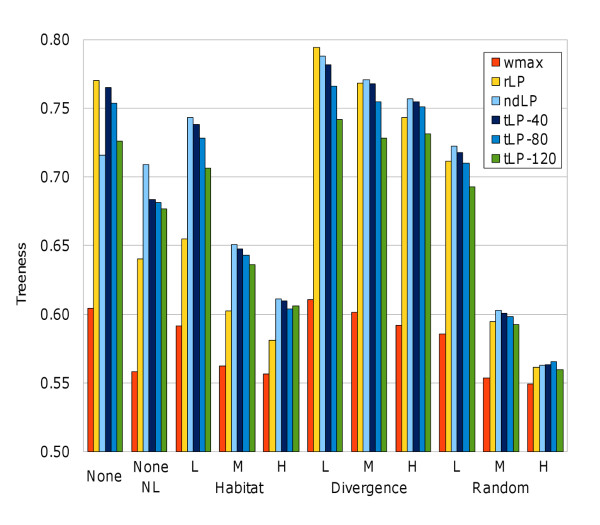
**Treeness scores for all combinations of simulation type and network inference method**. None = *NoLGT*, NL = No loss, L = low, M = medium, H = high.

The normalized treeness scores *T_norm _*ranged between 0.549 and 0.794 across all combinations of simulated LGT regime and network reconstruction method (Figure 1 and Supporting Table S1 in Additional File 2). Although *T_norm _*varied across different approaches, the non-LP *wmax *approach always yielded the lowest treeness scores (0.549 ≤ *T_norm _*≤ 0.611). In addition to comparing raw treeness scores, we also used the distribution of 50 or 250 *wmax *scores as the basis for paired-sample *t*-test comparisons against the LP-based techniques, with a null hypothesis of no difference between *wmax *and the LP alternative. Of the eleven different rate/regime combinations, the removal of dominated proteins (*ndLP*) yielded the best treeness score in eight cases, with reweighting (*rLP*) best in two of the remaining cases, and thresholding at an e-value cutoff of 1.0 × 10^-80 ^(*tLP-80*) best in one case. In general the *tLP-T *strategies yielded slightly smaller *T_norm _*scores, with the progressive decrease from *T *= 40 to *T *= 120 suggesting that the genes removed due to thresholding did contain a small additional amount of information about genomic affinities. In ten out of eleven cases, the *ndLP *treeness scores were significantly better than the *wmax *scores, with maximal improvements (corrected *p *= 1.21 × 10^-14^, the minimum possible value retrievable from R after Bonferroni correction) in cases where the tree signal was strong due to minimal LGT (*noLGT *and all low levels of LGT), or LGT that reinforces the vertical signal in the tree (medium and high rates of divergence-biased LGT). The *noLGT-noLoss *simulations provided a curious exception to this rule; since duplication and loss were the only phenomena that distinguished these simulations from the *noLGT *runs, it may be that the presence of paralogous sequences in the phylogenetic profile matrix actually reinforced the treelike signal. Improved recovery of treelike signals under divergence-biased regimes of LGT has been seen before [23], with preferential exchange between close relatives reinforcing their overall similarity. The *T_norm _*of all approaches decreased in cases where LGT did not explicitly reinforce the reference tree signal, with the effect more pronounced under random LGT than under habitat-directed LGT (since habitat-directed LGT will to some extent preferentially occur among closely related genomes that share the same habitat due to common ancestry).

**Figure 2:**
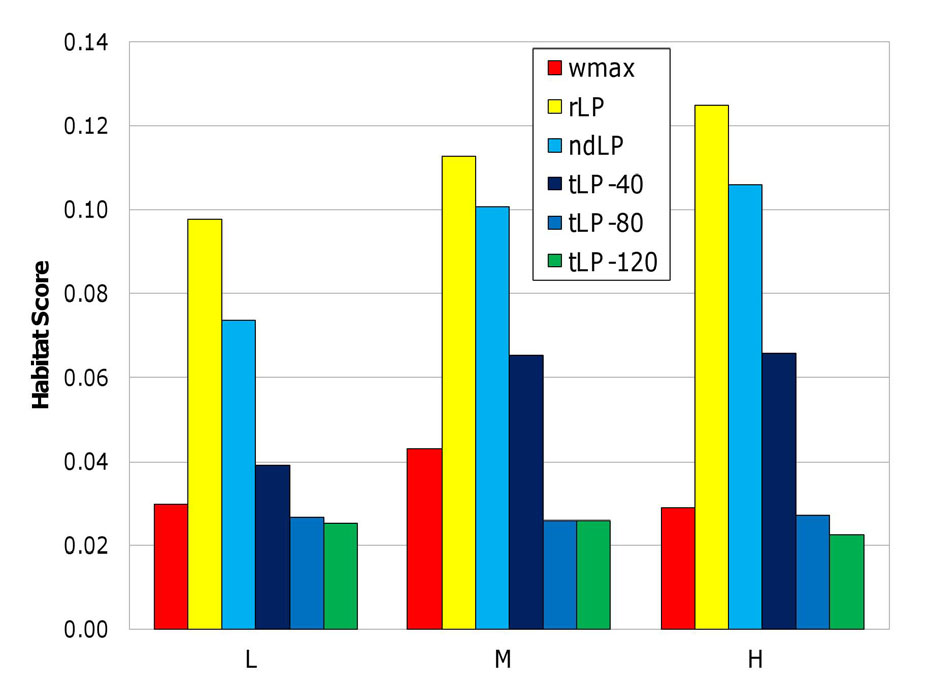
**Habitat scores for simulations carried out with habitat-restricted LGT under different network inference methods**. L = low, M = medium, and H = high rates of LGT.

Although the performance of *ndLP*, *tLP-T *and *rLP *was roughly similar in most simulations, *rLP *performed considerably worse in four cases: the three habitat-restricted LGT scenarios, and the unusual *noLGT-noLoss *simulation. It is unclear why *rLP *performed poorly (although still significantly better than *wmax*: corrected *p *= 2.96 × 10^-5^) in the latter case, but inspection of the habitat scores (Figure 2, and Supporting Table S2 in Additional File 2) showed that reduced treeness in *habLGT *simulations was due to more-accurate recovery of habitat-driven relationships. The habitat scores were significantly better than the *wmax *baseline for *rLP *(corrected *p *between 1.42 × 10^-5 ^and 9.17 × 10^-12^) and *ndLP *(corrected *p *between 1.36 × 10^-3 ^and 1.2 × 10^-7^), with no improvement seen in any of the *tLP-T *analyses. In both the *rLP *and *ndLP *cases, the habitat score increased with increasing rates of LGT, successfully capturing the greater contribution of biased LGT to the composition of genomes under these simulation conditions. Based on these results, *rLP *and *ndLP *give results that are somewhat complementary, with *rLP *capturing a broader range of patterns and *ndLP *focusing on those patterns that are strongest in the data.

**Figure 3:**
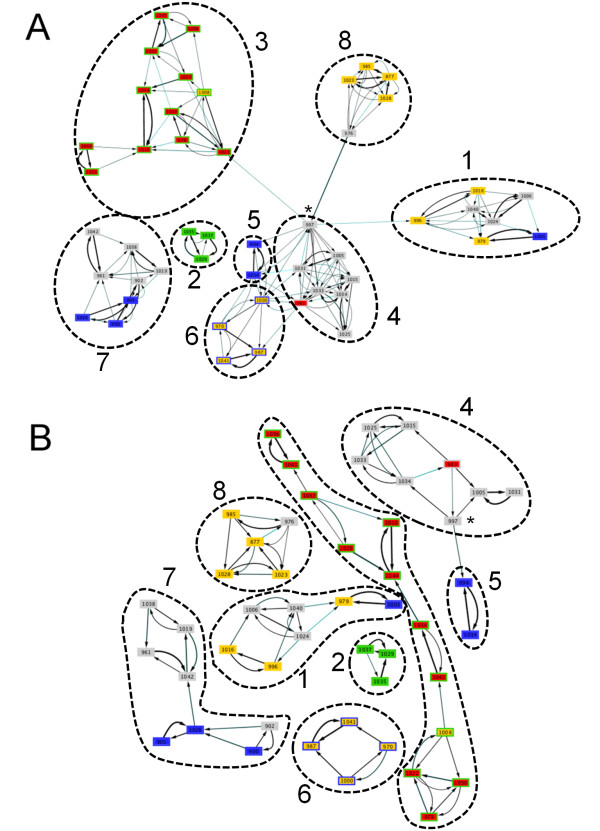
**Network of genome affinities and underlying speciation tree for one replicate of the *noLGT *simulation**. The network is based on the genome tree shown in Figure S2, using the *wLP *(panel A) and *ndLP *(panel B) methods. The edge color represents the distance in the tree measure, where black represents a distance of 0, and lighter colors represent larger distances up to 9 (lightest blue). The edge widths represent the degree of affinity between two nodes, corresponding to the weights obtained from the LP solution. Clades described in the main text are indicated with numbers 1-8, and genome 997 is highlighted with an asterisk.

**Figure 4:**
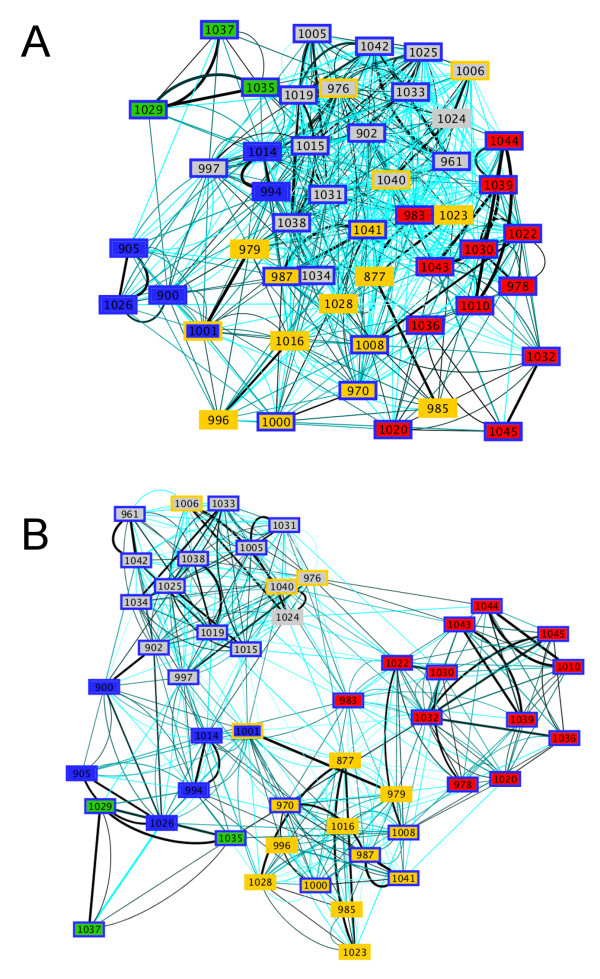
**Network of genome affinities and underlying speciation tree for one replicate of the *habLGT-high *simulation**. The network is based on the genome tree shown in Figure S1, using the *wLP *(panel A) and *ndLP *(panel B) methods. The edge color represents the distance in the tree measure, where black represents a distance of 0, and lighter colors represent larger distances up to 9 (lightest blue). The edge widths represent the degree of affinity between two nodes, corresponding to the weights obtained from the LP solution.

To further investigate the relationship between the *rLP *and *ndLP *approaches, we chose one simulation run and visualized the corresponding species tree (Supporting Figure S2 in Additional File 1) and constructed graphs for the *noLGT *(Figure 3) and *habLGT-high *(Figure 4) simulations. The graphs recovered in the absence of LGT are disconnected, with the *rLP *approach producing three components and 174 links (Figure 3A) and *ndLP *yielding seven components and 94 links (Figure 3B). In both networks, each genome is connected to at least one genome in the group that is a sister to it. But the *rLP *graph has many more connections both within and between clades: in the *rLP *case, clades 1, 3, 4, 5, 6 and 8 identified in Figure S1 are connected to one another, mostly through the early-branching "lonely lineage" 997. This genome also links clades 4 and 5 in the *ndLP *graph, although all other connections are lost. Within each identified clade (except clade 5 which contains only two genomes), the number of connections dropped by 28%-56%. The connections lost were predominantly more-distant ones, with the average phylogenetic distance between connected genomes dropping by approximately half.

The habitat-restricted LGT simulations yielded connected graphs with many more links: 669 when *rLP *was used for reconstruction (Figure 4A), and 440 when *ndLP *was used (Figure 4B). The automated layout performed by Cytoscape [40] suggests good separation of genomes based on shared habitats, with higher apparent separation achieved by the *ndLP *solution. Only 22.4% (*rLP*) and 35.9% (*ndLP*) of links were between members of the same clade, as compared with 89.0% (*rLP*) and 98.9% (*ndLP*) of links inferred from the *noLGT *simulation. In assessing the influence of shared habitats, we distinguish between the *current *habitat of each genome (represented by the colored borders in Figures S2, 3, and 4) and the *habitat of longest residence *(represented by the colored rectangles) for each genome. Two genomes currently occupying the same habitat may have many opportunities to share genes, but if one of the two genomes was until recently present in a different habitat, and was present in that habitat for a long time, then its genomic composition may still be more reflective of its former habitat. Although, as indicated above, fewer than 36% of links were to members of the same clade, a great many links could be attributed to shared habitats: in the *rLP *network reconstruction, 47.2% of links were between genomes sharing the same current habitat, and 49.8% of links were between genomes with the same habitat of longest residence. The numbers for *ndLP *were even higher: 65.7% of links were between genomes in the same current habitat, and 74.5% were between genomes sharing the same habitat of longest residence. Most strikingly, between 49% and 62% of these links were between members of the same habitat, but of *different *clades. This demonstrates the ability of the *rLP *and *ndLP *approaches to recover complementary vertical and lateral evolutionary signals.

The proportion of the original protein data set retained by the *ndLP *strategy under different simulation conditions was itself informative about the extent of discordant phylogenetic signal (Supporting Table S3 in Additional File 2). The largest proportion of proteins (>80%) were removed in the datasets simulated without LGT. Among LGT-containing datasets, divergence-biased LGT had the highest rate of removal (70-77%), while approximately 66% of proteins were removed from the datasets simulated with random LGT. Low rates of habitat-restricted LGT yielded similar removal rates, but removal decreased for medium (62.2%) and high (22.5%) rates of LGT. Protein removal therefore appears to correlate with the expected strength of relationships that are in conflict with the species tree: against a baseline established by random LGT, divergence-biased LGT has a higher rate of removal, while habitat-restricted LGT has a much lower rate. Given the apparent complementarity of the *rLP *and *ndLP *approaches, we applied these two techniques to the inference of evolutionary patterns from microbial genomic data sets.

### Affinities of *Aquifex aeolicus *and other thermophiles

Although *Aquifex aeolicus *is considered to be among the earliest-diverging bacteria on the basis of 16S rDNA phylogeny, phylogenomic analyses have identified a number of alternative partner lineages, including the Epsilon-proteobacteria and various Archaeal lineages [5,24]. Lateral genetic transfer appears to have played an important role in the evolution of bacterial thermophily and hyperthermophily [41,42], and the identification of partner lineages, as well as the different types of molecular functions shared with these lineages, can indicate the route by which *A. aeolicus *became a hyperthermophile.

**Figure 5:**
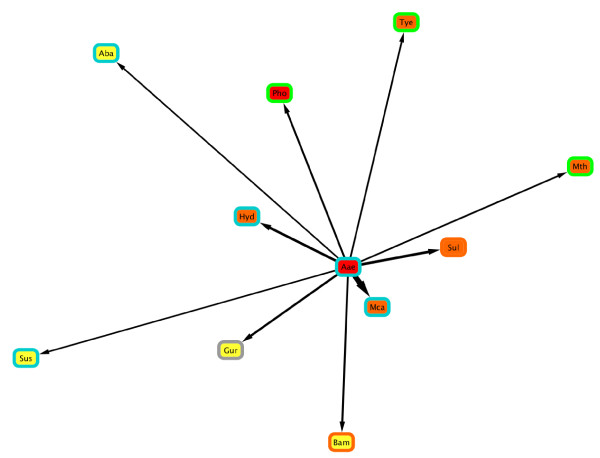
**Genomes comprising the *ndLP *solution in a comparison of 865 genomes against *A. aeolicus***. Edge thicknesses are proportional to the corresponding weight assigned to each genome in the *ndLP *solution. Each genome's oxygen usage is shown with the border color of its corresponding node (light blue = aerobic, orange = anaerobic, gray = facultative aerobe), and its temperature preference with the node color (red = hyperthermophilic, orange = thermophilic, yellow = mesophilic).

We first performed a comparison of 865 sequenced genomes covering 34 distinct phyla against *A. aeolicus*, to determine which of these had strong affinities. A total of ten genomes from five phyla were retained in the *ndLP *solution (Figure 5, and Supporting Table S4 in Additional File 2). Two types of organisms are represented in the *A. aeolicus *solution: other thermophiles and hyperthermophiles, and very large genomes. Included among the thermophiles are the other two sequenced representatives of phylum Aquificae, *Hydrogenobaculum *sp. Y04AAS1 and *Sulfurihydrogenibium *sp. YO3AOP1; two Archaea including a methanogen and *Pyrococcus horikoshii*; *Thermodesulfovibrio yellowstonii*, a hot spring bacterium from phylum Nitrospirae; and a rare thermophilic proteobacterium. Mesophilic genomes in the solution set originate from the Acidobacteria and Proteobacteria and include the giant (~10 megabase) genome of the acidobacterium *Solibacter usitatus *and one member of the Delta-proteobacteria, a group often implicated in LGT with other phyla [43,44]. Consistently with our benchmarking results above, the *rLP *solution returned a much larger number of genomes (29 as opposed to ten): of these, none was shared between the two lists but two genera (*Geobacter *and *Pyrococcus*) were represented in both solutions. Interestingly, while both other Aquificae were missing from the *rLP *solution, *Thermosipho melanesiensis *and *Nitratiruptor *sp. SB155-2 which represent alternative affinities for the Aquificae lineage were both present. Many of the other genomes in the *rLP *solution set were also thermophiles or hyperthermophiles from groups including Crenarchaeota, Euryarchaeota, Dictyoglomi and Clostridia.

We repeated the *ndLP *analysis for both of the other Aquificae in our set, and recovered solution sets consisting of 14 genomes (*Hydrogenobaculum*: Supporting Table S5 in Additional File 2) and ten genomes (*Sulfurihydrogenibium*: Supporting Table S6 in Additional File 2). Like the *A. aeolicus ndLP *solution, both the *Hydrogenobaculum *and *Sulfurihydrogenibium *lists contained the two non-self Aquificae genomes. Beyond this, however, no single genome appeared in all three solution sets, although all three solutions contained representatives from the Delta-proteobacteria (either *Geobacter*, *Anaeromyxobacter*, or both). The *Hydrogenobaculum *set included five representatives of phylum Chlorobi and two thermophilic Chloroflexi, all other non-Aquificae genomes belonging to the Proteobacteria including two methylotrophs and an acidophile. Linkages to Chlorobi, Chloroflexi and other genomes may reflect LGT related to hot, acidic habitats or to the use of particular compounds as sources of energy and nutrients. Genomes in the *Sulfurihydrogenibium *solution set include several soil bacteria and bacteria with large genomes (e.g., *Burkholderia cenocepacia *J2315, > 8 Mb in size). In addition to the two Aquificae, two extremophiles are present in the set: *T. yellowstonii *and a halophilic euryarchaeote.

**Figure 6:**
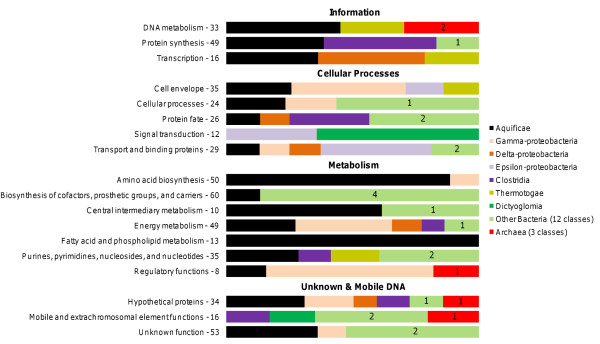
**Taxonomic breakdown of *ndLP *solutions across 18 TIGR role categories**. The width of each colored bar indicates the proportional contribution of the relevant taxonomic class to the overall *ndLP *solution. The number of proteins retained in each category is indicated after the category name, and the number of aggregated classes is indicated when Archaea or 'other' Bacteria constitute part of a solution.

Different functional classes of proteins show different propensities toward LGT [5,45], and are thus likely to show different taxonomic affinities if LGT is sufficiently frequent. We explored this question for *A. aeolicus *by repeating the *ndLP *analysis on subsets of genes, grouped by TIGR Role Category http://cmr.jcvi.org/cgi-bin/CMR/shared/RoleList.cgi. Since these optimizations are performed on distinct subsets of the full dataset, genomes appearing in the full solution may not appear in the functional subset solutions, and vice versa. Figure 6 shows the breakdown of LP solutions for each category. The Aquificae are represented in 16 of 18 categories, and represent the entire solution in the 'fatty acid and phospholipid metabolism' category. Within the informational categories, DNA metabolism is dominated by hyperthermophilic Thermotogae and Archaea, possibly reflecting the effects of recent transfer to assist in stabilization of the bacterial chromosome. The solution for the 'protein synthesis' category includes two thermophiles: the actinobacterium *Rubrobacter xylanophilus *and *Symbiobacterium thermophilum*, a member of class Clostridia. While protein synthesis genes are thought to be particularly recalcitrant to LGT, the alternative affinities here may be due to aminoacyl-tRNA synthetases and other proteins that do not form part of large complexes [46]. Transcriptional proteins are associated with Thermotogae and two Delta-proteobacteria in the *ndLP *solution. Cellular process proteins are less closely tied to the Aquificae: other important groups include the Epsilon-proteobacteria (especially the thermal vent bacterium *Nitratiruptor *sp. SB155-2), Gamma-proteobacteria, and the Alpha-proteobacteria *Rhodopseudomonas palustris *and *Sphingopyxis alaskensis*, which constitute > 50% of the solution in the 'Cellular process' category. Metabolic enzymes show evidence of heterogeneous origins as well, particularly those involved in cofactor and carrier biosynthesis, which include genomes from phyla Cyanobacteria (*Thermosynechococcus elongatus*), Chlorobi (*Chlorobium tepidum *and *Chloroherpeton thalassium*), Flavobacteria (*Flavobacterium johnsoniae*) and Nitrospirae (*T. yellowstonii*) in their *ndLP *solution set. Not surprisingly, mobile elements and hypothetical proteins show a great deal of heterogeneity as well, including contributions from thermophilic Archaea, Clostridia, Cyanobacteria, and Dictyoglomi, as well as a number of mesophiles from across several phyla.

The *A. aeolicus *solutions indicated a strong contribution of genetic material from other thermophilic and hyperthermophilic lineages. But the broader picture of thermophile evolution may involve gene sharing relationships that do not directly implicate the Aquificae. To further investigate the relationships among thermophiles, we inferred an all-versus-all *ndLP *network for the 80 out of 865 genomes in our set that were labeled as thermophilic or hyperthermophilic, which included 48 Bacteria from 12 phyla, and 32 Archaea from four phyla. From this set we removed one genome from each of two named species (*Streptococcus thermophilus *and *Thermus thermophilus*) that were represented twice. Six phyla (Chlorobi, Deinococcus-Thermus, Korarchaeota, Nanoarchaeota, Nitrospirae, and Verrucomicrobia) had only a single thermophilic or hyperthermophilic representative, while the Firmicutes (18 genomes from classes Bacilli and Clostridia), Crenarchaeota (16 genomes) and Euryarchaeota (14 genomes) were the largest sets.

**Figure 7:**
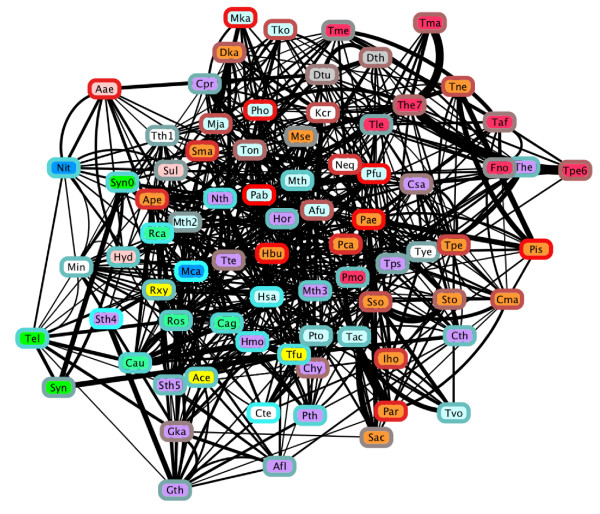
***ndLP *graph for 78 thermophilic and hyperthermophilic organisms**. Nodes are colored by phylum, with white used to indicate the six phyla with only a single representative (see text). Node borders indicate OGT of the associated organism, ranging from cyan = 45°C (*S. thermophilus*, Sth4) to red = 103°C (*Pyrococcus abyssi*).

The resulting *ndLP *graph (Figure 7) is connected, with an average node (in + out) degree of 8.68 indicating that genomes are connected to 11.3% of other members of the set, on average. Nodes with a low in-degree have few genomes contributing to their overall solution; these genomes typically had close relatives (congeners) present in the data set. For example, *Thermotoga petrophila *(in-degree: 1) and *Thermotoga maritima *(in-degree: 2) had large incoming weights from other members of genus *Thermotoga*. The solution for *Synechococcus *sp. JA-2-3B'a included a closely related strain of the same genus, and the actinobacterium *Thermobifida fusca*. The maximum in-degree of any vertex in the graph was 15, which was observed for *M. capsulatis *(covering nine distinct phyla), *Petrotoga mobilis *(seven distinct phyla), *Moorella thermoacetica *(nine distinct phyla), and *Candidatus *"Korarchaeum cryptofilum" (six distinct phyla). Each of these organisms is among the most taxonomically distinct in its group, with *K. cryptophilum *the lone representative of phylum Korarchaeota. Genomes with a large out-degree are significant contributors to the thermophilic gene pool in other organisms: out-degrees in the graph ranged from 0 (*Nanoarchaeum equitans *and *S. thermophilus*) to 30 (*Halobacterium salinarum*; ten distinct phyla). *A. aeolicus *has an in-degree of seven and an out-degree of four: affinities with its sister lineage *Hydrogenobaculum *are retained, but the elimination of the large, mesophilic genomes that were present in Figure 5 introduces new thermophiles into its solution set, including *Nitratiruptor *and organisms from phyla Dictyoglomi, Crenarchaeota and Firmicutes. No edges connect genomes from phyla Aquificae to Thermotogae or vice versa.

The thermophiles and hyperthermophiles in our dataset cover a range of optimal growth temperatures (OGT) from 45°C to 103°C. We considered whether affinities were stronger between genomes with similar temperature preferences relative to a null model by considering the weight and temperature differential associated with each edge. Summing the product of edge weight and OGT difference for each of the 677 edges in our graph, we obtained a score of 9822.5. Performing the same summation with randomized genomic OGTs yielded an average score of 12,334.6, with a standard deviation of 471.7. The *p*-value of 6.40 × 10^-8 ^associated with the resulting Z-score strongly suggests that genomes with similar OGT tend to have stronger associations in the LP solution. To remove the effect of taxonomic similarity from this result, we next considered only those edges that connected members of different phyla. The *p*-value in this case was several orders of magnitude larger (0.002) but still supported a stronger association between genomes with similar OGT.

### Vertical and lateral signal in genus *Pseudomonas*

*Pseudomonas *is a highly diverse genus within the Gamma-proteobacteria, comprising > 120 named species with a wide diversity of lifestyles, including saprophytes, plant, fungal and animal pathogens, and marine and soil organisms [47]. This diversity is supported by large genome sizes and the ready acquisition of plasmids and genomic islands [48]. In the case of *Pseudomonas aeruginosa*, pathogenicity and resistance traits can also be rapidly acquired due to the presence of integrons [49]. *Pseudomonas*-associated mobile elements can have very broad host ranges, raising the possibility of significant genetic flux between members of this genus and other groups of microorganisms. Indeed, the taxonomic affinities of open reading frames in *P. aeruginosa *islands are extremely broad, mapping to a wide range of phyla [50].

**Figure 8:**
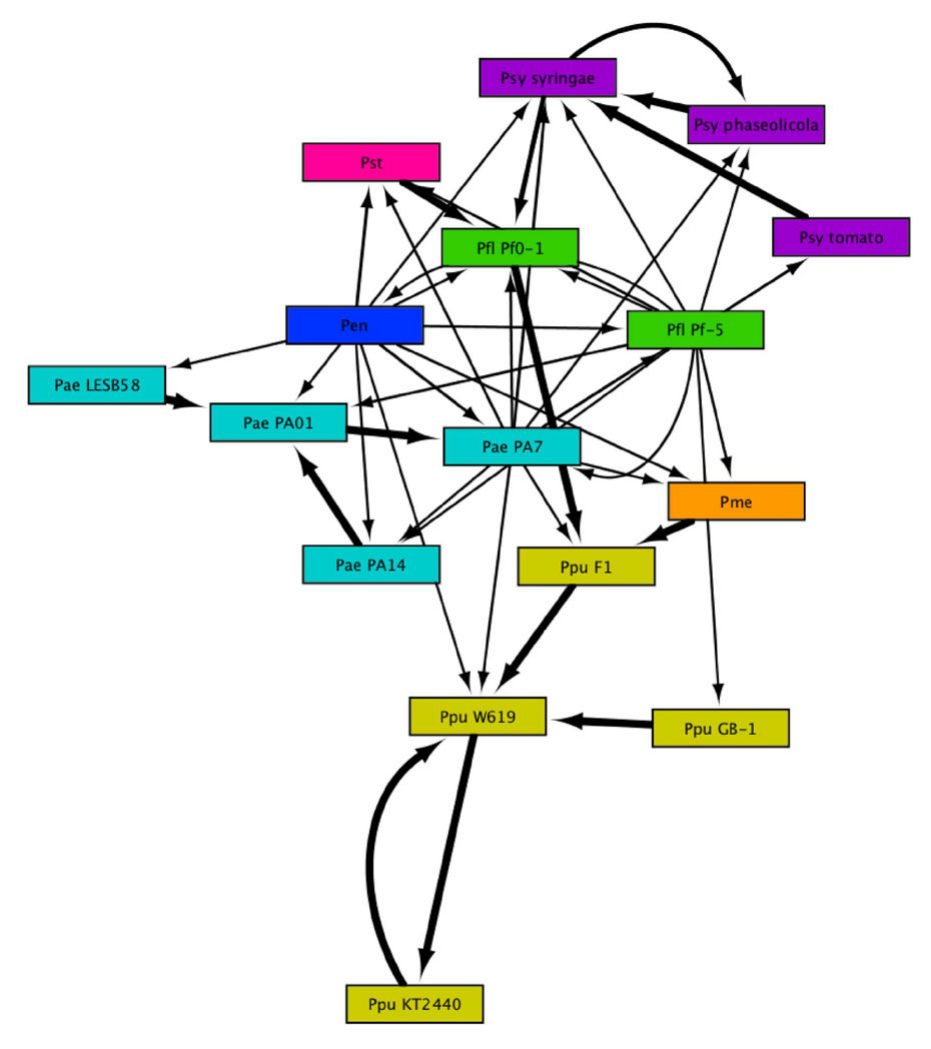
***ndLP *graph for 16 genomes from genus *Pseudomonas*, with colors used to distinguish species**. Abbreviations used in this graph are defined in Table 1.

To assess the relative influence of within- and between-species gene flow, we constructed a network based on *ndLP *weights between 16 different members of genus *Pseudomonas *covering a total of seven named species (Table 1 and Supporting Figure S3 in Additional File 1). Although Table 1 indicates the primary ecological motivations for studying the various species of *Pseudomonas*, the niche range of members of this genus tends to be very broad and has not been fully mapped for many of the listed isolates, so the full extent of ecological overlap between the various species and strains cannot be precisely quantified. Figure 8 shows the connected graph of *Pseudomonas *genomes based on the *ndLP *solution. A total of 45 edges connect the 16 nodes in the graph, yielding an average node degree (incoming + outgoing edges) of 2.81. Each genome is therefore connected (in the undirected sense) to 17.6% of the other members of the data set. A considerable amount of vertical signal is captured by the graph, with members of the same species typically connected by edges with strong weights. The exception to this trend is *P. fluorescens*; although these genomes are weakly connected to one another, strain Pf0-1 has a strong connection to *P. putida *F1, and strain Pf-5 has connections to every other named species except *P. entomophila*. Two of the three species with a single representative (*P. stutzeri *and *P. mendocina*) are strongly connected to one other genome, while *P. entomophila *has affinities to all six other species in the set. The difference in connectivity patterns may relate to the status of *P. stutzeri *and *P. mendocina *as the smallest genomes (4.6 and 5.1 Mbp, respectively) in our data set, while *P. entomophila *is considerably larger at 5.9 Mbp. The relatively small genomes and few affinities may also reflect reduced interactions with eukaryotic hosts (as epiphytes, pathogens, etc.) relative to other *Pseudomonas *species, diminishing the importance of host-adaptive LGT.

**Table 1:** Pseudomonas genomes used in this analysis.

Name	Size	Prot	Pl	Primary description	Abbreviation
***P. aeruginosa *LESB58**	6.6	5925	0	Epidemic strain	Pae LESB58

***P. aeruginosa *PA7**	6.6	6446	0	Non-respiratory	Pae PA7

***P. aeruginosa *PAO1**	6.3	5742	0	Lab strain, moderately virulent	Pae PAO1

***P. aeruginosa *UCBPP PA14**	6.5	6039	0	Highly virulent	Pae PA14

***P. entomophila *L48**	5.9	5274	0	Insect pathogen, orally ingested	Pen

***P. fluorescens *Pf0-1**	6.4	5852	0	Saprophyte, promotes plant nutrition	Pfl Pf0-1

***P. fluorescens *Pf-5**	7.1	6270	0	Saprophyte, promotes plant nutrition	Pfl Pf-5

***P. mendocina *ymp**	5.1	4782	0	Bioremediation of toluene	Pme

***P. putida *F1**	6	5494	0	Saprophyte, bioremediation	Ppu F1

***P. putida *GB-1**	6.1	5611	0	Saprophyte, bioremediation	Ppu GB-1

***P. putida *KT2440**	6.2	5501	0	Saprophyte, bioremediation	Ppu KT2440

***P. putida *W619**	5.8	5389	0	Saprophyte, bioremediation	Ppu W619

***P. stutzeri *A1501**	4.6	4219	0	Nitrogen fixation	Pst

***P. syringae *pv. Phaseolicola 1448A**	5.9	5511	2	Plant pathogen	Psy phaseolicola

***P. syringae *syringae B728a**	6.1	5258	0	Plant pathogen	Psy syringae

***P. syringae *tomato str. DC3000**	6.4	5796	2	Plant pathogen	Psy tomato

Size in megabases, number of predicted proteins (Prot), and the number of plasmids (Pl) are indicated.

We extended this core network by including comparisons of all 16 Pseudomonads against the other 849 genomes in our data set. All possible pairwise comparisons between *Pseudomonas *genomes were performed, but other genomes were compared only to members of this genus, and not to each other. The *ndLP *solution includes 196 non-*Pseudomonas *genomes (23.1% of the original dataset) of which 157 are other Proteobacteria. Apart from the Chlorobi (3/11 genomes retained), no phylum with > 2 representative genomes had more than 25% of its genomes retained. Major underrepresented groups included the Actinobacteria (6/57 = 10.5% retained), Firmicutes (12/145 = 8.3% retained), Archaea (2/55 = 3.6% retained), and eukaryotes (1/46 = 2.2% retained). There is a clear preference for proteobacterial genomes in the *ndLP *solution: over 64% of Beta-proteobacterial genomes were included in the solution, primarily from order Burkholderiales. Within the Gamma-proteobacteria, over 50% of orders Alteromonadales, non-*Pseudomonas *Pseudomonadales, Vibrionales, and Xanthomonadales were retained. Most of the included Alpha-proteobacteria were soil organisms from classes Caulobacterales, Rhizobiales, Rhodobacterales, and Rhodospirillales.

**Figure 9:**
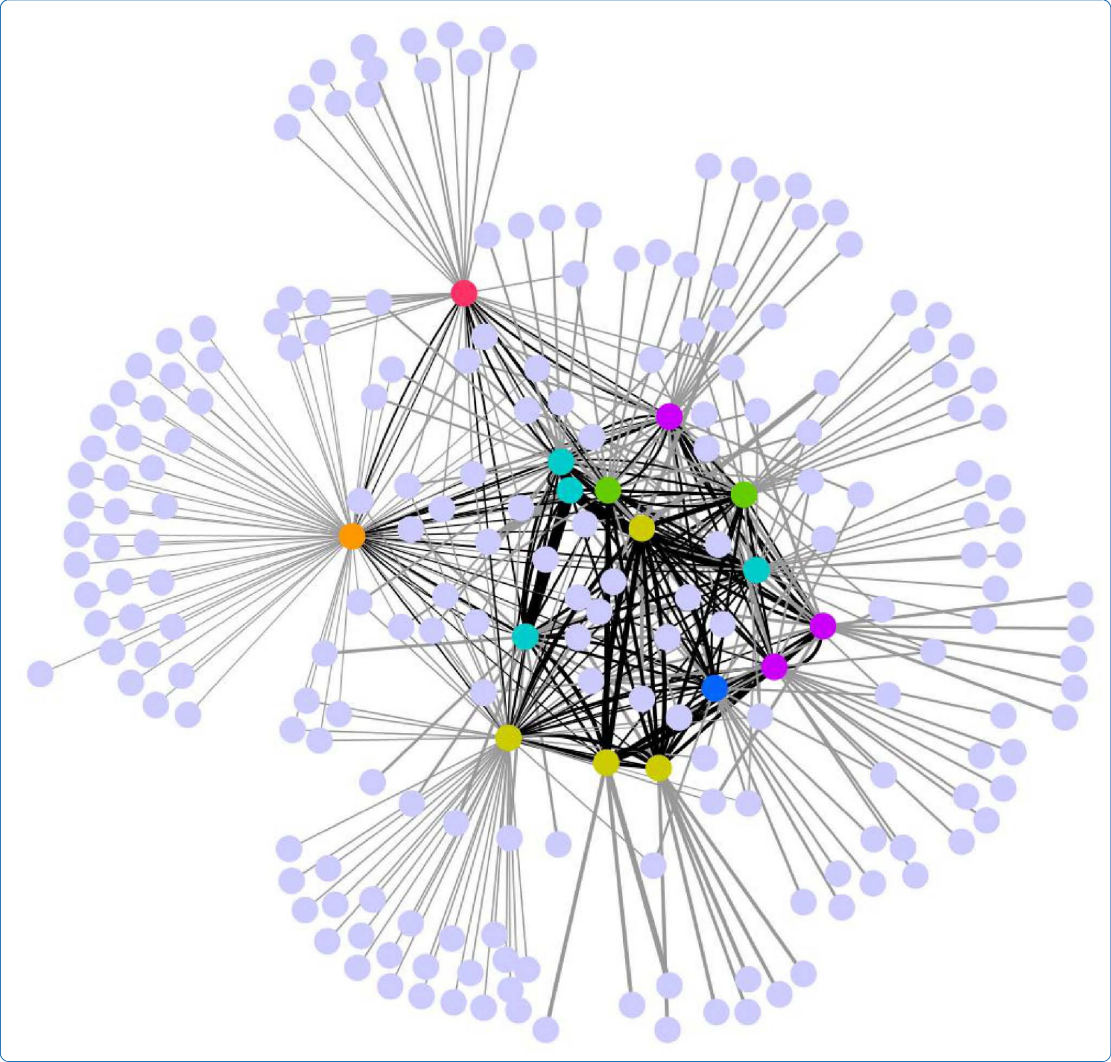
***ndLP *Network of genome affinities recovered from a comparison of 865 sequenced genomes against the 16 members of *Pseudomonas***. Nodes corresponding to *Pseudomonas *genomes are colored as in Figure 8; all other genomes all colored in gray.

**Figure 10:**
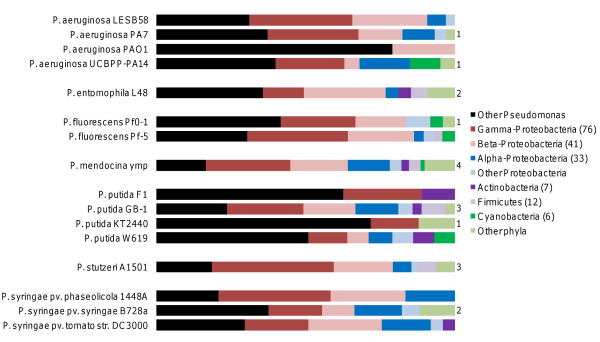
**Taxonomic breakdown of *ndLP *solutions for each of 16 *Pseudomonas *genomes**. The width of each colored bar represents the proportional contribution of the relevant taxonomic group to the overall *ndLP *solution. Numbers next to 'other phyla' bars indicate the number of other phyla contained within this solution.

The most striking feature of the *Pseudomonas ndLP *graph (Figure 9) is the presence of 'plumes' of non-*Pseudomonas *genomes that are matched by only a single genome within the genus. Taxonomic summaries of connecting genomes are shown in Figure 10. The most highly connected genome is that of *P. mendocina*, which is linked to a total of 66 non-*Pseudomonas *genomes; 35 of these are connected to no other *Pseudomonas *genome and therefore constitute its plume of unique connections. Groups that are particularly prominent in the *P. mendocina *solution include orders Burkholderiales (12 genomes), Rhizobiales (6 genomes), and Alteromonadales (5 genomes). Mobile elements and the ~200 hypothetical proteins with no match to other Pseudomonads likely account for many of the connected genomes. *P. stutzeri *has connections to 33 other genomes including 13 in its plume, which includes genomes of the root-associated organisms *Bacillus pumilus *and *Sinorhizobium meliloti*, other plant-associated bacteria such as *Xanthomonas oryzae *and *Burkolderia thailandensis*, and several marine bacteria. Its shared affinities comprise additional nitrogen-metabolising genomes such as *Azoarcus *sp. BH72 and *Nitrosomonas eutropha *and several additional marine bacteria. The last uniquely represented species in our dataset, the insect pathogen *P. entomophila*, connects to 15 genomes of which five are not connected to any other *Pseudomonas *genome. Its plume covers five different phyla and includes *Aspergillus fumigatus*, the only eukaryotic genome in the solution set; *Clostridium botulinum*; *Photobacterium profundum*; *Frankia *sp. Ccl3; and *Gramella forsetii*. Of these, *A. fumigatus *and *C. botulinum *have pathogenic potential, while *G. forsetii *is able to degrade large organic compounds. Its other partners include nitrogen metabolizers and pathogens.

Genomes from the same species differed considerably of the strength of their connections. The most dramatic example of this was seen in *P. aeruginosa *PAO1, which connected to only four other genomes: the other three strains of *P. aeruginosa *and the metabolically diverse *Ralstonia eutropha*. This dearth of connections may reflect the high degree of similarity between strain PAO1 and its conspecific isolates, as well as the fact that the genome of strain PAO1 is 200-300 kb smaller than the other *P. aeruginosa *genomes. Other *P. aeruginosa *genomes match pathogens including *Serratia proteamaculans*, *Neisseria meningitidis *and various enteric bacteria, but include a wide range of environmental bacteria as well. *P. putida *GB-1 has connections to 50 other non-*Pseudomonas *genomes: like *P. mendocina*, many of the connections are to Burkholderiales and Rhizobiales, with strong representation from the Enterobacteriales and genera *Acinetobacter *and *Geobacter*. At the other extreme of connectivity, *P. putida *KT2440 connects only to *Chlorobium chlorochromatii*, *Halorhodospira halophila*, and *Salmonella enterica*. Similarly, *P. putida *F1 connects to only four non-*Pseudomonas *genomes, from genera *Aeromonas*, *Proteus*, *Xanthomonas *and *Streptomyces*. Contrary to the general tendencies of larger genomes to have more connections, strains KT2440 and F1 are the largest and third-largest members of their group. The remaining species, *P. fluorescens *and *P. syringae*, had similar distributions of affinities to environmental and pathogenic bacteria.

## Discussion

We have developed and validated an LP approach to the reconstruction of genomic affinities. Based on the EvolSimulator results reported above, these LP approaches (particularly the *rLP *and *ndLP *variants we focus on) are better able to recover strong, distinctive vertical and lateral inheritance patterns than are network approaches that make direct use of BLASTP similarities. The LP solution can be used to generate graphs of genomes, which carry some advantages relative to genome phylogeny-based approaches. Many genome phylogeny approaches generate partitionings of taxa which are visualized as branches and intersecting sets of parallel lines. Such graphs are typically subject to a circular compatibility or similar constraints [51,52], which complicates the representation of long-distance transfer relationships. Newer approaches such as cluster networks can circumvent these limitations by adding reticulate nodes, which are not constrained by phylogenetic distance, to a tree, but these approaches are NP-complete and have thus far been applied to a relatively small number of gene sets [53]. Unlike the above approaches, which are explicitly phylogenetic in nature, our approach relies on the inference of directed edges and edge weights by the LP solver to generate a graphical representation of genomic affinities. This approach mirrors most closely the approaches of Lima-Mendez et al. [29] and Halary et al. [31] mentioned in the Introduction, but the filtering of data and inference of weighted, directed edges by our LP approach yields more information about the magnitude and direction of transfer.

The LP solution is based on the calculation of weights for different genomes; for a given target genome X, a large weight attached to a comparison genome Y_j _indicates that Y_j _is an important part of the solution. This importance, however, does not necessarily correlate with the number of genes in X that appear to originate from Y_j_; rather, if Y_j _has a small number of genes that are very similar to genes in X, and which cannot easily be accounted by other genomes in the set, then a high weight for Y_j _is likely to be recovered in the LP solution. Very recent LGT events will tend to be highlighted by this approach, since the resulting similarity profile will be distinct and the affinity (BLAST e-value) extremely high. The removal of dominated strategies further accentuates this effect, since genes whose inheritance is mostly vertical and that therefore have 'canonical' patterns of similarity to other genomes will tend to be removed *en masse *due to the presence of highly conserved proteins that follow the same pattern of similarity and have lower BLAST expectation values. Although the use of our approach inevitably filters out some lateral relationships among the genomes under consideration, filtering is necessary in any approach to try and distinguish signal from noise. Splits graphs are often controlled by restricting the dimensionality of the solution, imposing a uniform constraint on all relationships without regard to the number of affinities within any particular set of genomes [54]. Our approach has the capacity to return many connections where appropriate, as demonstrated in our analyses of data sets with high levels of LGT.

Our case studies illustrated particular strengths of the method, as well as highlighting challenges in the interpretation of the results. The analysis of *A. aeolicus *returned a plausible set of genomes, although the exclusion of groups such as Thermotogae and Epsilon-proteobacteria from the main solution set (both groups were represented in the role category breakdown solution set) was surprising. It is likely that at least some of the proteins previously observed to support these affinities are also present in *Hydrogenobaculum *and *Sulfurihydrogenibium*, thus making these other Aquificae the natural partners for *A. aeolicus *and eliminating the distinctive contributions of the other two groups. The presence of large genomes in the solution set suggests that large, versatile genomes may act as hubs in the global LGT network [55], since so many of the genes they possess can be adaptive in different environments. Although we made no effort to correct for sampling effort in different groups, most of the global and functional results appeared to be driven by genuine affinities rather than raw counts of sequenced genomes. For instance, while the heavily sequenced Gamma-proteobacteria were indeed present in the role category solution sets of *A. aeolicus *(Figure 6), their contribution was limited to a few categories and was similar in importance to much smaller groups such as the Epsilon-proteobacteria. Conversely, Proteobacteria dominated the *Pseudomonas *solution set, with substantial contributions from soil bacteria, marine bacteria and some pathogens. Although it is difficult to extract ecological narratives from large lists of genomes, in a broad sense these habitats are consistent with the primary known roles of members of genus *Pseudomonas*. The high degree of heterogeneity seen in the solution sets of some genomes (particularly *P. mendocina*) is interesting and worthy of further exploration using more-explicit phylogenomic techniques.

An important challenge in interpreting the results of an LP analysis is the construction of an appropriate summary of all genomes in the solution set. This was more straightforward for *A. aeolicus *than for genus *Pseudomonas*. When a particular group such as the Burkholderiales is assigned a large weight in a solution set, we can appreciate that this group may have made important genetic contributions to our genomes of interest. But genomes encode a complex set of processes, and the most reasonable hypothesis for a given connection such as the exchange of pathogenesis genes between *Escherichia coli *and *P. aeruginosa *may not be the correct one; genetic affinities may instead reflect gene transfers related to respiration, metabolism of available compounds, or other cellular processes. Also, transfers of genetic material that produce an edge in the LP graph may in fact have no adaptive role, having arisen as a consequence of random integration and rapid but not immediate turnover of introgressed genes, or due to the activity of phages, plasmids and other mobile elements. One way to address the question of which genomes have contributed which functions is to carry out a refined analysis as shown in Figure 6; a set of such optimizations can reveal which functions can be attributed to which originating taxonomic groups. Another approach is to refer back to the original set of genes, either through inspection of the BLAST tables and comparison of affinities, or by performing a phylogenomic analysis to identify the affinities of genes in the genome of interest. In such a situation the LP optimization can play the additional role of filtering the initial set of genomes: for example, in the *Pseudomonas ndLP *analysis we reduced the candidate set of 'interesting' genomes from 849 to 203, which would accelerate subsequent steps of orthologous set inference, sequence alignment and phylogenetic analysis. Another alternative we did not explore here is the pooling of genomes within a particular species or genus prior to performing the LP analysis; such an approach would simplify the solution (for example, by representing genus *Burkholderia *as a single node in the *Pseudomonas *solution above) while still showing key affinities among the genomes of interest.

In this work we used BLAST results as the raw material, without attempting to define orthologs and paralogs, to infer genomic relationships. The technique could also be applied to orthologous gene profiles generated using a technique such as COG [56], or to structural indices that can potentially identify homologous genes that are too distantly related to be detected by BLAST. The efficient nature of the LP solver suggests that much larger data sets can be handled by this technique once the appropriate input affinity data have been generated.

## Conclusions

The intergenomic relationships among microbes are a complex mix of vertical signal and recent and ancient LGT events. Our approach does not aim to describe the full set of evolutionary relationships among a set of genomes, but instead emphasizes a genome's closest genetic neighbours. Recent events may reflect adaptive changes that are most relevant to an organism's current ecological range and interactions with other organisms. Challenges remain in understanding which optimization technique is most appropriate to the question at hand, and how complex sets of intergenomic affinities should be interpreted. Future work will aim to refine the optimization techniques to improve their robustness and make the interpretation of connection weights more explicit. An important challenge that is not specific to our approach lies in the use of affinity graphs or networks to explore ecological and evolutionary hypotheses. Microorganisms have complex sets of ecological attributes, and in many cases similarities of organisms in their habitat range, uptake of mobile elements, and tolerance of different environmental conditions cannot be precisely quantified. While it is possible to draw some conclusions from the topology of affinity graphs and the functional categories associated with different edges in the graph, more-detailed experimental characterization of microorganism ranges will ultimately be needed to enable a deeper understanding of intergenomic affinities.

## Methods

### Datasets and functional annotations

Simulated datasets were constructed using EvolSimulator 2.1.0 [39]. EvolSimulator allows replicated analyses to be carried out, with most parameters of a run including species tree topology and gene mutation and substitution parameters kept constant across multiple runs, but with variable rates and regimes of gene gain, gene loss, and LGT. Identical random number seeds and similar configuration files were used for all runs in a given replicate: for example, the '-s' and '-z' seeds which control stochastic events in gene and genome evolution respectively, were set to 1248706227 and 1649402786 for the first replicate. The configuration files used for all 11 EvolSimulator runs are available as supporting text in Additional File 3. Each simulation was performed for 2500 iterations, with the speciation/extinction probability set to 0.7. The maximum number of lineages was set to 50. Genomes in each run were assigned to one of five habitats, with the probability of habitat migration set to 0.0015 per iteration, with the consequence that most genomes had between 0 and 5 habitat changes in their history.

All simulated genes had a length of 900 nucleotides, with an average mutation rate of 0.01 substitutions per site per iteration. In the *noLGT-noLoss *simulation, genome size was kept constant at 800 genes, whereas in the other 10 simulations genome size was allowed to drift (via gene gains, losses, and LGT where appropriate) between 500 and 1000 genes. Genome size could increase or decrease by a minimum of 0 and a maximum of 5 genes per iteration. Three underlying rates (25, 250, and 2500 LGT events per iteration) and three regimes (unconstrained, divergence-restricted, and habitat-restricted LGT) were combined to yield a total of nine runs with simulated LGT events. The rate of LGT specifies the average (drawn from a Poisson distribution) number of *proposed *LGT events between donor-recipient pairs for a given iteration: this is potentially problematic because all proposed events are successful in the unconstrained regime, whereas proposed events may be rejected due to excessive genome divergence or habitat incompatibility in the other two regimes. To avoid mismatches in the number of successful events for a given underlying rate of LGT, we used the '-retryLGT = 100' argument to EvolSimulator, which proposes another donor/recipient pair if a proposed LGT event is rejected due to regime-specific constraints, up to a maximum of 100 times. Consequently the *number *of LGT events should be comparable across regimes for a given fixed rate, but the *distribution *of donor/recipient pairs will differ. In the case of divergence-restricted LGT, the probability of success for a proposed transfer decreased linearly with increasing number of iterations separating two genomes (i.e., # of iterations since their last common ancestor) up to 500, and LGT forbidden between genomes whose common ancestor was > 500 generations in the past.

Gene sequences and habitat histories were output from EvolSimulator in GenBank and FASTA-formatted files, which were used for the subsequent analyses described below. Node distances used in the treeness and habitat scores (see Results) were computed from Newick-formatted files using the Bio::Phylo library of BioPerl http://search.cpan.org/dist/Bio-Phylo/. All statistical analyses were carried out using version 2.7.1 of the R software package http://r-project.org.

The 865 sequenced genomes used in this analysis were obtained from the National Center for Biotechnology Information (NCBI) via rsync on 28 November, 2008. The set included the genomes of 46 eukaryotes, 55 archaea and 764 bacteria. Conceptually translated open reading frames from each retrieved file were stored in a local relational database, and subjected to BLAST analysis as described in the following section. OGT data were retrieved from NCBI or from the primary literature if OGT data were unavailable at NCBI.

### Genome-scale sequence similarity analysis and graph construction

Simulated or predicted proteomes were subjected to all-versus-all BLASTP analysis using version 2.2.18 of the NCBI 'blastall' program. Parameters of the BLAST runs were chosen according to the recommendations of Moreno-Hagelsieb et al. [57], particularly the soft filtering of low-information segments using the -F "m S" option. The BLOSUM62 matrix was used to score local alignments, with gap opening and extension costs of 1 during the dynamic programming phase. The default choice of composition-based score adjustments (-T) was used.

All-versus-all BLASTP results were stored in database tables showing all query/subject pairs having an e-value of 0.001 or less. These were converted into the necessary format by identifying, for each gene *X_i _*in genome X, the best-matching protein *j *in genome *y_j_*, for all *y *in the set of genomes Y. The resulting table of e-values α contained one row for each *X_i_*, and one column for each *y_j_*, with α*_i,j _*the e-value of the best-matching protein. This matrix was transformed to matrix A as in equation (1). Any proteins that were identical across all genomes were removed from matrix A, since the presence of such proteins would lead to all possible LP solutions being equally good. This removal was performed on the *Pseudomonas *dataset; a total of 62 identical homologous sets of proteins were removed prior to the LP step.

Matrix A was used to construct the constraints in a LP problem, either by dividing the rows by the row sums (*rLP*), applying a threshold number to the matrix (*tLP-T*), or by removing all dominated rows (*ndLP*). The constraints were implemented in the GNU MathProg modeling language, described by equation (5), where the comparison genomes were variables to be optimized. The LP problems were solved using GNU linear programming kit (GLPK: http://www.gnu.org/software/glpk). Edge weights of the genome networks were determined by the activity on the genomes from the GLPK solution files. The total activity for each test genome was constrained to 1. All graphs were visualized in Cytoscape 2.7.0 [40]. Different layout algorithms were used for different visualizations: EvolSimulator reference trees were organized using the hierarchical layout algorithm, graphs centered on *A. aeolicus *with a force-directed layout, and more-highly-connected graphs (thermophiles and *Pseudomonas*) with an organic layout.

### *Pseudomonas *reference tree

We elected to use the RNA polymerase subunit beta (*rpoB*) gene as the reference for relationships among *Pseudomonas *genomes. *rpoB *has been used in such a way for other groups [58,59], and is expected to be less prone to LGT due to its participation in a multisubunit complex. Sixteen annotated *rpoB *gene sequences, one for each genome, were retrieved, and we retrieved the *rpoB *sequence from *Cellvibrio japonicus*, a member of the family Pseudomonadaceae, to serve as a putative outgroup. A multiple sequence alignment was built using FSA 1.14.5 [60], with default parameters and the '-nucprot' flag to align conceptual translations of the *rpoB *genes, rather than the raw DNA sequence, in order to preserve the correct reading frame in all sequences. A phylogenetic tree was constructed with RAxML 7.2.5 [61], with a general time reversible model of nucleotide substitution, eight discrete gamma rate categories, and an invariant category. One hundred fast bootstrap replicates were performed to assess the support for each internal node. The resulting tree was visualized using Dendroscope 2.7.4 [62].

## Authors' contributions

CH developed the LP approach and performed the experiments. Both authors made significant contributions to experimental design, analysis of results, and manuscript writing. Both authors read and approved the final manuscript.

## Supplementary Material

Additional file 1 **Supporting Figures S1-S3**. Supporting Figure S1. **Relationship between data set size and running time in seconds**. Times are shown for *rLP *(white circles) and *ndLP *(black circles). Supporting Figure S2. **Species tree showing the evolutionary relationships among all simulated genomes for one replicate EvolSimulator run**. Leaves represent extant genomes after 2500 simulation iterations. Colored borders indicate the current habitat occupied by each genome, while filled rectangles indicate the habitat occupied by that genome and its ancestors for the longest period of time during the 2500 simulated iterations. Clades described in the main text are indicated with numbers 1-8, and genome 997 is highlighted with an asterisk. Supporting Figure S3. **Phylogenetic tree of RNA polymerase beta subunit (RpoB) proteins from 16 genomes of genus *Pseudomonas***. Leaf labels correspond to abbreviations defined in Table 1. Numbers at each internal node represent the support for the implied partitioning of taxa from 100 bootstrap replicates. Click here for file

Additional file 2 **Supporting Tables S1-S6**. Supporting Table S1. **Treeness scores (T) for simulated data sets under different network reconstruction approaches**. Statistical significance is indicated using the exponent E of the Bonferroni-corrected p-value for the treeness statistic. Supporting Table S2. **Habitat scores (H) for simulated data sets under different network reconstruction approaches**. Statistical significance is indicated using the exponent E of the Bonferroni-corrected p-value for the treeness statistic. Supporting Table S3. **Removal of dominated proteins from simulated data sets**. For each combination of regime and rate, the proportion of all simulated proteins that were removed in the *ndLP *strategy is shown. Supporting Table S4. **Genomes included in the *ndLP *solution for *A. aeolicus***. For each genome, the domain (Dom: Bac = Bacteria, Arch = Archaea, phylum, class, species name, abbreviation (Abbr) in Figure 5, and weight (Wt) in the *ndLP *solution are shown. Supporting Table S5. **Genomes included in the *ndLP *solution for *Hydrogenobaculum *sp. Y04AAS1**. For each genome, the phylum, class,, and weight (Wt) in the *ndLP *solution are shown. All genomes in this solution are from domain Bacteria. Supporting Table S6. **Genomes included in the *ndLP *solution for *Sulfurihydrogenibium *sp. YO3AOP1**. For each genome, the phylum, class,, and weight (Wt) in the *ndLP *solution are shown. Apart from *H. marismortui *(Archaea), all genomes in this solution are from domain Bacteria. Click here for file

Additional file 3 **Supporting Text**. Supporting Text. Generic configuration file for EvolSimulator, with run-specific settings indicated at the end of the file. Click here for file
