# Supplementary figures and images for: Assembling networks of microbial genomes using linear programming
Source: BMC Evol Biol. 2010 Nov 20;10:360. doi: 10.1186/1471-2148-10-360 (PMC3224671; doi:10.1186/1471-2148-10-360)

Figure S1

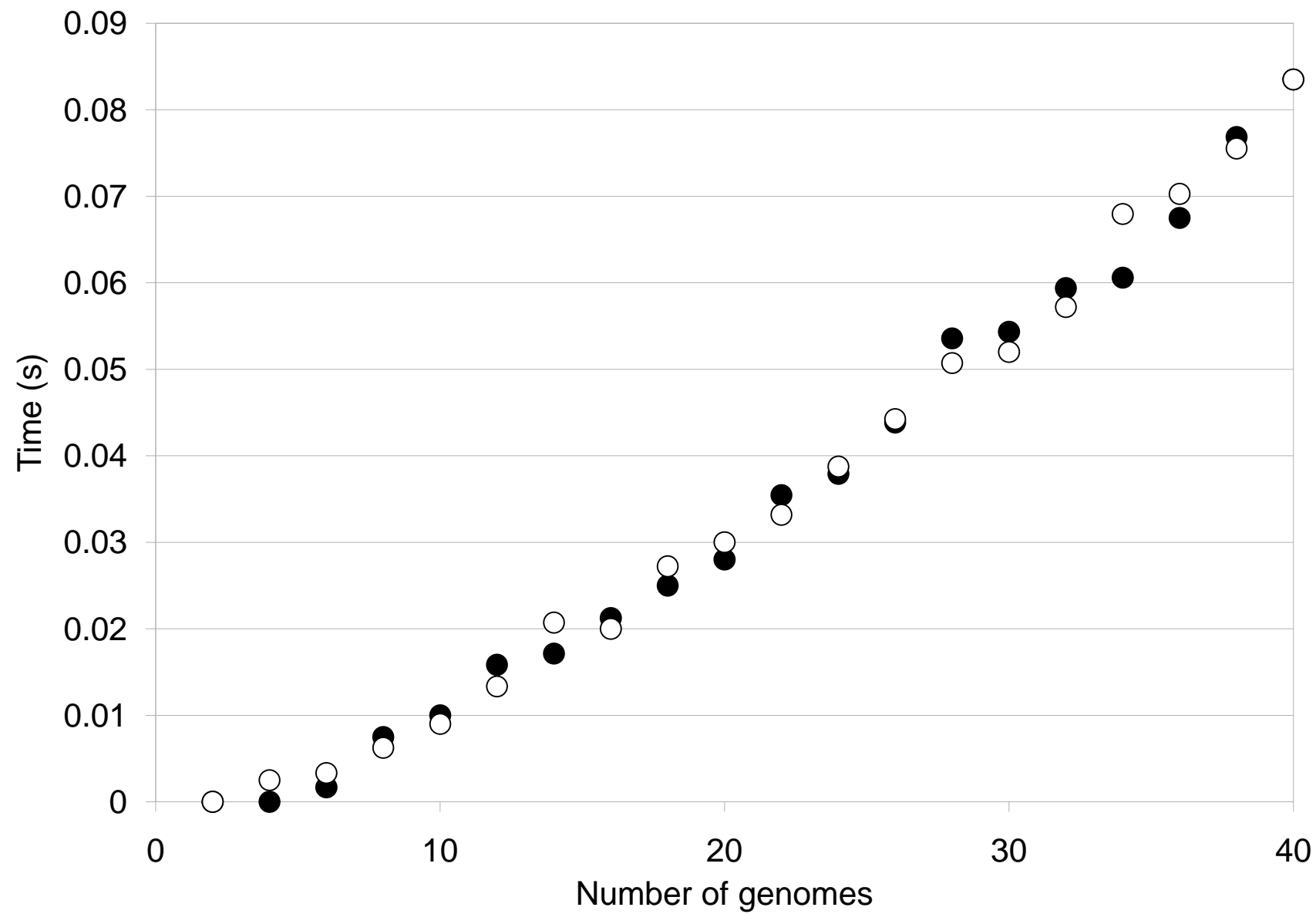

Figure S2

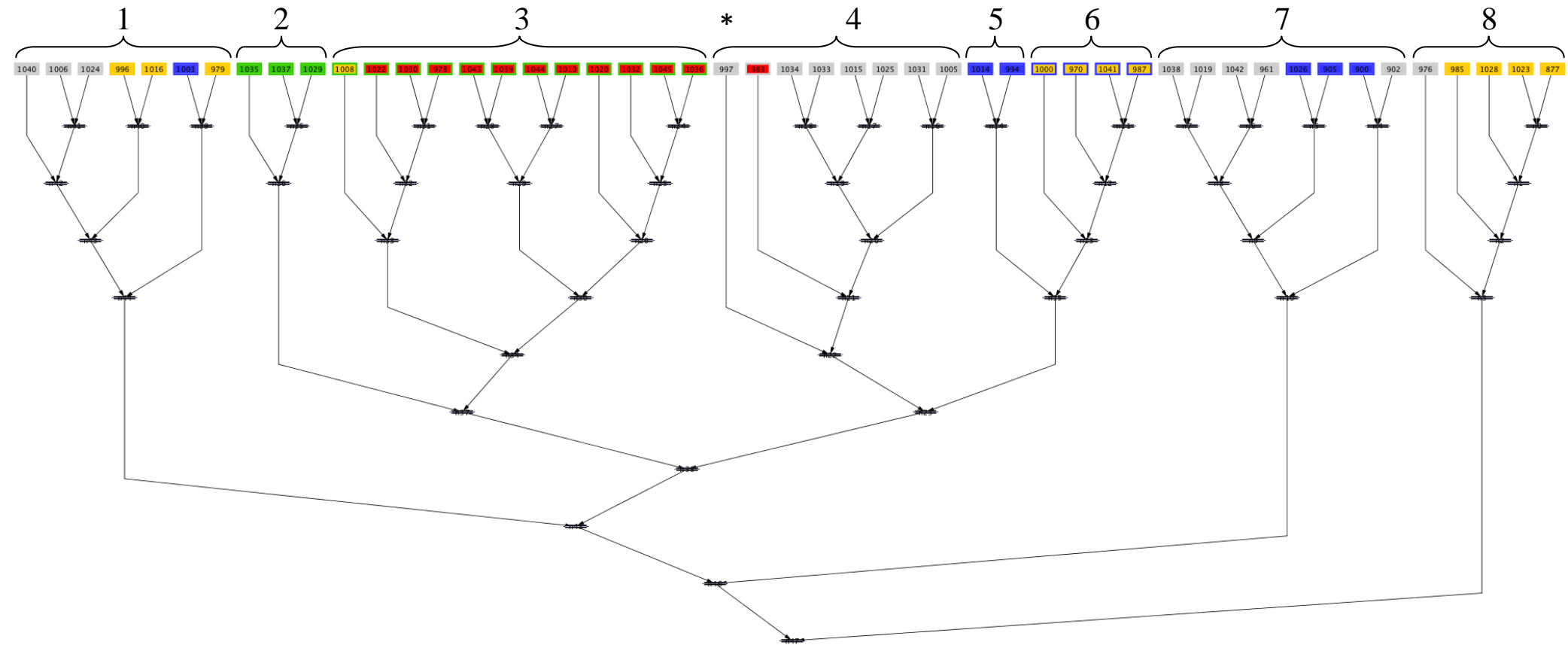

Figure S3

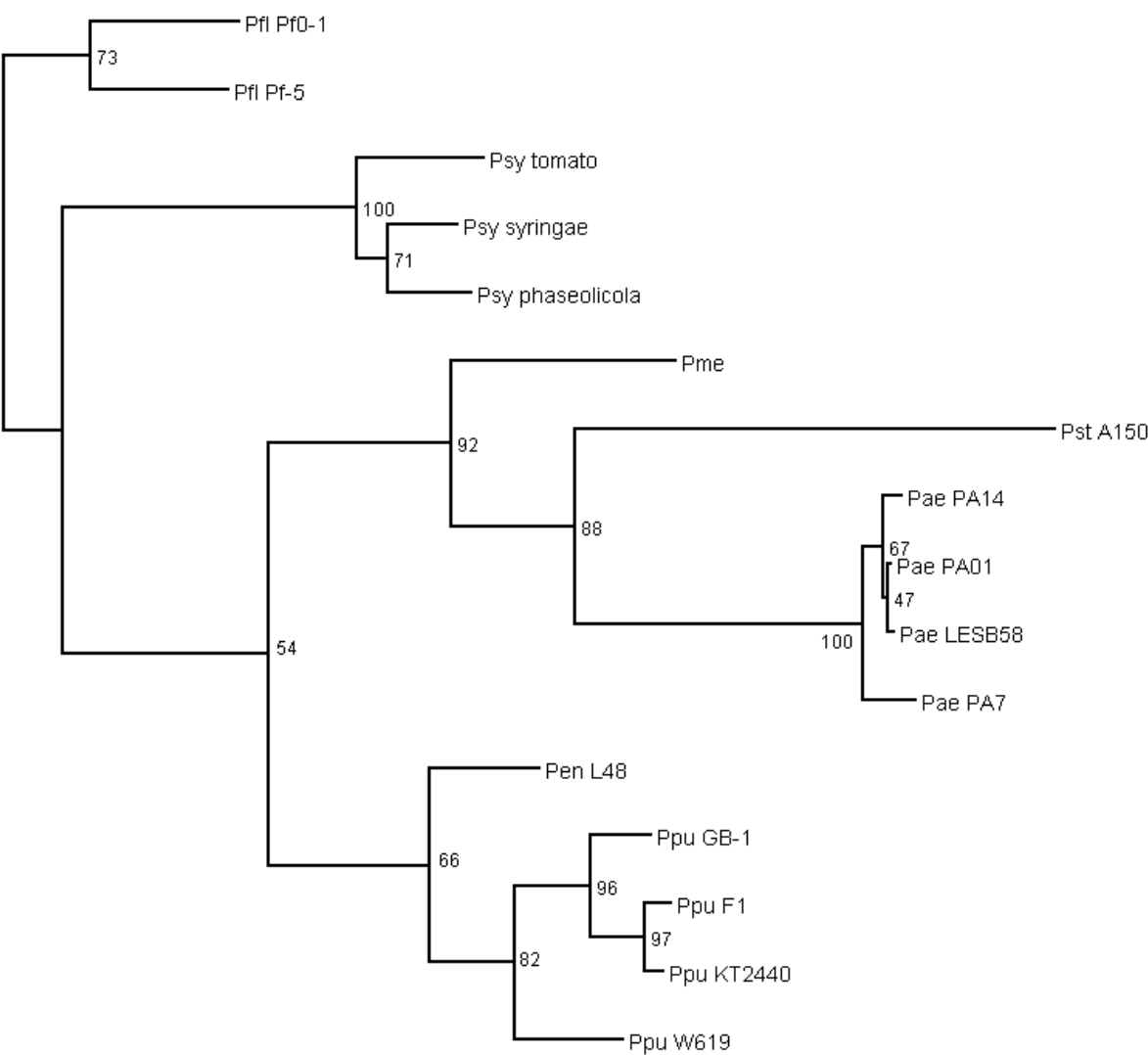

Supplement: Additional file 1 — Supporting Figures S1-S3. Supporting Figure S1. Relationship between data set size and running time in seconds. Times are shown for rLP (white circles) and ndLP (black circles). Supporting Figure S2. Species tree showing the evolutionary relationships among all simulated genomes for one replicate EvolSimulator run. Leaves represent extant genomes after 2500 simulation iterations. Colored borders indicate the current habitat occupied by each genome, while filled rectangles indicate the habitat occupied by that genome and its ancestors for the longest period of time during the 2500 simulated iterations. Clades described in the main text are indicated with numbers 1-8, and genome 997 is highlighted with an asterisk. Supporting Figure S3. Phylogenetic tree of RNA polymerase beta subunit (RpoB) proteins from 16 genomes of genus Pseudomonas. Leaf labels correspond to abbreviations defined in Table 1. Numbers at each internal node represent the support for the implied partitioning of taxa from 100 bootstrap replicates. [file 1471-2148-10-360-S1.PDF]
