# Supplementary material for: Assembling networks of microbial genomes using linear programming
Source: BMC Evol Biol. 2010 Nov 20;10:360. doi: 10.1186/1471-2148-10-360 (PMC3224671; doi:10.1186/1471-2148-10-360)
Supplement: Additional file 2 — Supporting Tables S1-S6. Supporting Table S1. Treeness scores (T) for simulated data sets under different network reconstruction approaches. Statistical significance is indicated using the exponent E of the Bonferroni-corrected p-value for the treeness statistic. Supporting Table S2. Habitat scores (H) for simulated data sets under different network reconstruction approaches. Statistical significance is indicated using the exponent E of the Bonferroni-corrected p-value for the treeness statistic. Supporting Table S3. Removal of dominated proteins from simulated data sets. For each combination of regime and rate, the proportion of all simulated proteins that were removed in the ndLP strategy is shown. Supporting Table S4. Genomes included in the ndLP solution for A. aeolicus. For each genome, the domain (Dom: Bac = Bacteria, Arch = Archaea, phylum, class, species name, abbreviation (Abbr) in Figure 5, and weight (Wt) in the ndLP solution are shown. Supporting Table S5. Genomes included in the ndLP solution for Hydrogenobaculum sp. Y04AAS1. For each genome, the phylum, class,, and weight (Wt) in the ndLP solution are shown. All genomes in this solution are from domain Bacteria. Supporting Table S6. Genomes included in the ndLP solution for Sulfurihydrogenibium sp. YO3AOP1. For each genome, the phylum, class,, and weight (Wt) in the ndLP solution are shown. Apart from H. marismortui (Archaea), all genomes in this solution are from domain Bacteria. [file 1471-2148-10-360-S2.PDF]

Supporting Table S1. **Treeness scores (T) for simulated data sets under different network reconstruction approaches.** Statistical significance is indicated using the exponent E of the Bonferroni-corrected p-value for the treeness statistic.

Regime	Rate	*wmax*	*rLP*		*ndLP*		*tLP-40*		*tLP-80*		*tLP-120*	
		T	*T*	*E*	*T*	*E*	*T*	*E*	*T*	*E*	*T*	*E*
**noLGT**	0	0.604	0.770	-14	0.716	-10	0.765	-14	0.754	-14	0.726	-11
**noLGT-NL**	0	0.558	0.640	-5	0.709	-9	0.683	-10	0.682	-10	0.677	-9
**divLGT**	Low	0.611	0.794	-14	0.788	-14	0.782	-14	0.766	-14	0.742	-14
**divLGT**	Medium	0.601	0.768	-14	0.771	-14	0.768	-14	0.755	-14	0.728	-14
**divLGT**	High	0.592	0.743	-14	0.757	-14	0.755	-14	0.751	-14	0.731	-14
**randLGT**	Low	0.586	0.712	-14	0.723	-14	0.718	-14	0.710	-14	0.693	-14
**randLGT**	Medium	0.553	0.595	-2	0.603	-3	0.601	-3	0.598	-3	0.592	-2
**randLGT**	High	0.549	0.561	-1	0.563	0	0.563	0	0.566	0	0.560	-1
**habLGT**	Low	0.592	0.655	-8	0.743	-14	0.738	-14	0.728	-14	0.706	-14
**habLGT**	Medium	0.562	0.602	-3	0.651	-9	0.648	-9	0.643	-8	0.636	-7
**habLGT**	High	0.557	0.581	-1	0.611	-5	0.610	-4	0.604	-2	0.606	-4

Supporting Table S2. **Habitat scores (H) for simulated data sets under different network reconstruction approaches.** Statistical significance is indicated using the exponent E of the Bonferroni-corrected p-value for the treeness statistic.

**Rate**	***wmax***	***rLP***		***ndLP***		***tLP-40***		***tLP-80***		***tLP-120***	
	H	*H*	*E*	*H*	*E*	*H*	*E*	*H*	*E*	*H*	*E*
**low**	0.297	0.978	-4	0.736	-2	0.392	0	0.267	0	0.254	0
**medium**	0.431	1.127	-6	1.008	-5	0.654	-1	0.259	0	0.259	0
**high**	0.289	1.250	-10	1.060	-6	0.659	-1	0.272	0	0.224	0

Supporting Table S3. **Removal of dominated proteins from simulated data sets.** For each combination of regime and rate, the proportion of all simulated proteins that were removed in the *ndLP* strategy is shown.

LGT Regime	LGT Rate	Proportion Removed
**noLGT**	0	0.811
**noLGT-noLoss**	0	0.919
**divLGT**	low	0.760
**divLGT**	medium	0.705
**divLGT**	high	0.764
**randLGT**	low	0.662
**randLGT**	medium	0.656
**randLGT**	high	0.656
**habLGT**	low	0.660
**habLGT**	medium	0.622
**habLGT**	high	0.225

Supporting Table S4. **Genomes included in the *ndLP* solution for *A. aeolicus*.** For each genome, the domain (Dom: Bac = Bacteria, Arch = Archaea, phylum, class, species name, abbreviation (Abbr) in Figure 5, and weight (Wt) in the *ndLP* solution are shown.

**Dom**	**Phylum**	**Class**	**Species**	**Abbr**	**Wt**
Arch	Euryarchaeota	Methanobacteria	*Methanothermobacter thermautotrophicus* str. Delta H	Mth	0.037
Arch	Euryarchaeota	Thermococci	*Pyrococcus horikoshii* OT3	Pho	0.060
Bac	Acidobacteria	Acidobacteria	*Acidobacteria* bacterium Ellin345	Aba	0.022
Bac	Acidobacteria	Solibacteres	*Solibacter usitatus* Ellin6076	Sus	0.021
Bac	Aquificae	Aquificae	*Hydrogenobaculum* sp. Y04AAS1	Hyd	0.121
Bac	Aquificae	Aquificae	*Sulfurihydrogenibium* sp. YO3AOP1	Sul	0.132
Bac	Nitrospirae	Nitrospira	*Thermodesulfovibrio yellowstonii* DSM 11347	Tye	0.037
Bac	Proteobacteria	Beta	*Burkholderia ambifaria* AMMD	Bam	0.067
Bac	Proteobacteria	Delta	*Geobacter uraniireducens* Rf4	Gur	0.088
Bac	Proteobacteria	Gamma	*Methylococcus capsulatus* str. Bath	Mca	0.414

Supporting Table S5. **Genomes included in the *ndLP* solution for *Hydrogenobaculum* sp. Y04AAS1.** For each genome, the phylum, class,, and weight (Wt) in the *ndLP* solution are shown. All genomes in this solution are from domain Bacteria.

Phylum	Class	Species	Wt
Aquificae	Aquificae	*Aquifex aeolicus* VF5	0.043
Aquificae	Aquificae	*Sulfurihydrogenibium* sp. YO3AOP1	0.075
Chlorobi	Chlorobia	*Chlorobaculum parvum* NCIB 8327	0.178
Chlorobi	Chlorobia	*Chlorobium tepidum* TLS	0.104
Chlorobi	Chlorobia	*Chlorobium phaeobacteroides* DSM 266	0.171
Chlorobi	Chlorobia	*Chlorobium phaeovibrioides* DSM 265	0.038
Chlorobi	Chlorobia	*Prosthecochloris aestuarii* DSM 271	0.075
Chloroflexi	Chloroflexi	*Chloroflexus aggregans* DSM 9485	0.077
Chloroflexi	Chloroflexi	*Chloroflexus aurantiacus* J-10-fl	0.073
Proteobacteria	Alpha	*Methylobacterium nodulans* ORS 2060	0.013
Proteobacteria	Alpha	*Methylobacterium populi* BJ001	0.024
Proteobacteria	Alpha	*Acidiphilium cryptum* JF-5	0.073
Proteobacteria	Delta	*Anaeromyxobacter dehalogenans* 2CP-C	0.029
Proteobacteria	Gamma	*Hahella chejuensis* KCTC 2396	0.026

Supporting Table S6. **Genomes included in the *ndLP* solution for *Sulfurihydrogenibium* sp. YO3AOP1.** For each genome, the phylum, class,, and weight (Wt) in the *ndLP* solution are shown. Apart from *H. marismortui* (Archaea), all genomes in this solution are from domain Bacteria.

Phylum	Class	Species	Wt
Aquificae	Aquificae	*Aquifex aeolicus* VF5	0.025
Aquificae	Aquificae	*Hydrogenobaculum* sp. Y04AAS1	0.181
Euryarchaeota	Halobacteria	*Haloarcula marismortui* ATCC 43049	0.175
Nitrospirae	Nitrospira	*Thermodesulfovibrio yellowstonii* DSM 11347	0.080
Proteobacteria	Beta	*Burkholderia cenocepacia* J2315	0.095
Proteobacteria	Delta	*Geobacter bemidjiensis* Bem	0.007
Proteobacteria	Delta	*Anaeromyxobacter* sp. Fw109-5	0.130
Proteobacteria	Epsilon	*Sulfurovum* sp. NBC37-1	0.227
Spirochaetes	Spirochaetes	*Leptospira biflexa* serovar Patoc strain Patoc 1	0.013
Verrucomicrobia	Opitutae	*Opitutus terrae* PB90-1	0.067
